# Estimating Disability Prevalence Among Adults by Body Mass Index: 2003–2009 National Health Interview Survey

**DOI:** 10.5888/pcd9.120136

**Published:** 2012-12-27

**Authors:** Brian S. Armour, Elizabeth Courtney-Long, Vincent A. Campbell, Holly R. Wethington

**Affiliations:** Author Affiliations: Elizabeth Courtney-Long, Vince Campbell, Holly R. Wethington, Centers for Disease Control and Prevention, Atlanta, Georgia.

## Abstract

**Introduction:**

Obesity is associated with adverse health outcomes in people with and without disabilities; however, little is known about disability prevalence among people who are obese. The purpose of this study was to determine the prevalence and type of disability among obese adults in the United States.

**Methods:**

We analyzed pooled data from sample adult modules of the 2003–2009 National Health Interview Survey (NHIS) to obtain national prevalence estimates of disability, disability type, and obesity by using 30 questions that screened for activity limitations, vision and hearing impairment, and cognitive, movement, and emotional difficulties. We stratified disability prevalence by category of body mass index (BMI, measured as kg/m^2^): underweight, less than 18.5; normal weight, 18.5 to 24.9; overweight, 25.0 to 29.9; and obese, 30.0 or higher.

**Results:**

Among the 25.3% of adult men and 24.6% of women in our pooled sample who were obese, 35.2% and 46.9%, respectively, reported a disability. In contrast, 26.7% of men and 26.8% women of normal weight reported a disability. Disability was much higher among obese women than among obese men (46.9% vs 35.2%, *P* < .001). Movement difficulties were the most common disabilities among obese men and women, affecting 25.3% of men and 37.9% of women.

**Conclusion:**

This research contributes to the literature on obesity by including disability as a demographic in characterizing people by body mass index. Because of the high prevalence of disability among those who are obese, public health programs should consider the needs of those with disabilities when designing obesity prevention and treatment programs.

## MEDSCAPE CME

Medscape, LLC is pleased to provide online continuing medical education (CME) for this journal article, allowing clinicians the opportunity to earn CME credit.

This activity has been planned and implemented in accordance with the Essential Areas and policies of the Accreditation Council for Continuing Medical Education through the joint sponsorship of Medscape, LLC and Preventing Chronic Disease. Medscape, LLC is accredited by the ACCME to provide continuing medical education for physicians. 

Medscape, LLC designates this Journal-based CME activity for a maximum of 1 **AMA PRA Category 1 Credit(s)™**. Physicians should claim only the credit commensurate with the extent of their participation in the activity.

All other clinicians completing this activity will be issued a certificate of participation. To participate in this journal CME activity: (1) review the learning objectives and author disclosures; (2) study the education content; (3) take the post-test with a 70% minimum passing score and complete the evaluation at www.medscape.org/journal/pcd (4) view/print certificate.

**Release date: December 26, 2012; Expiration date: December 26, 2013**

### Learning Objectives

Upon completion of this activity, participants will be able to:

- Assess the risk of disability associated with obesity
- Distinguish the most common type of basic actions difficulty among obese adults
- Distinguish the most common type of complex activity limitation among obese adults
- Evaluate the relationship between underweight status and disability

**EDITORS**

Rosemarie Perrin, Editor; Camille Martin, Editor, Preventing Chronic Disease. Disclosure: Rosemarie Perrin and Camille Martin have disclosed no relevant financial relationships.

**CME AUTHOR**

Laurie Barclay, MD. Freelance writer and reviewer, Medscape, LLC. Disclosure: Laurie Barclay, MD, has disclosed no relevant financial relationships.

**AUTHORS AND CREDENTIALS**

Disclosures: Brian Armour, PhD; Elizabeth Courtney-Long, MA, MSPH; Vince Campbell, PhD; Holly R. Wethington, PhD have disclosed no relevant financial relationships.

Affiliations: Brian Armour, Division of Human Development and Disability, Centers for Disease Control and Prevention, Atlanta, Georgia; Elizabeth Courtney-Long, Vince Campbell, Holly R. Wethington, Centers for Disease Control and Prevention, Atlanta, Georgia.

## Introduction

More than one-third of adults in the United States are obese, defined as having a body mass index (BMI, kg/m^2^) of 30 or higher (1). Understanding the reason for increased obesity prevalence is a public health issue, and reducing obesity prevalence is a public health policy challenge.

Disability affects more than 50 million people in the United States (2), and annual health care expenditures associated with disability approach $400 billion (3). Obesity is one of the leading secondary conditions (potentially preventable health problems that occur after the acquisition of a primary disability) among people with a disability (4,5). Among people with disabilities, obesity can lead to additional health problems, exacerbate existing health problems, and limit physical activity, thereby increasing the severity of disability (6).

The prevalence of obesity can vary by type of disability (4). Although information on the prevalence of chronic health conditions by weight status exists, information on the prevalence of reported disability among people who are obese is limited (7). Furthermore, there are no published reports based on US data describing the association of BMI with type of disability (8). According to the Centers for Disease Control and Prevention, new surveillance information is essential for monitoring progress in obesity prevention activities and for evaluating the effectiveness of interventions (9). By using data from the National Health Interview Survey (NHIS), we estimated the prevalence and type of disability among adults by BMI category.

## Methods

### Data sources

We obtained the data for this study from the 2003–2009 NHIS, a nationally representative, in-person, household survey of the civilian, noninstitutionalized US population that is conducted by the National Center for Health Statistics. The survey collects comprehensive demographic, health, behavioral risk, preventive health, and disability data. The NHIS data are used to examine trends in health and disability and to assess progress in meeting national health objectives (eg, Healthy People 2020).

The NHIS core consists of 4 major components: household, family, sample child, and sample adult. The household component collects demographic information on all household residents. The family component collects additional demographic information on each household family member, as well as data on health status, limitations, injuries, health care access and use, health insurance, and income and assets. For each family, 1 adult and 1 child are randomly selected, and more detailed information on specific conditions and health behaviors is collected. Our study used data from the 2003–2009 sample adult, family, and household questionnaires. NHIS complies with Department of Health and Human Services regulations (45 CFR 46) for protection of human subjects (http://www.cdc.gov/nchs/nhis.htm).

### Obesity definition

We used responses to 2 questions from the 2003–2009 NHIS questionnaires to determine BMI: “How tall are you without shoes?” and “How much do you weigh without shoes?” We used the NHIS definitions to categorize adults as underweight (BMI <18.5), normal weight (BMI, 18.5-24.9), overweight (BMI, 25−29.9), or obese (BMI ≥30) (1).

### Disability definition

Disability is a complex, multidimensional experience characterized by the interaction of an impairment (eg, spinal cord injury) with environmental factors (eg, lack of sidewalks) that may produce varying degrees of limitation in a person’s activities or participation in social activities (5,6,10,11). Disability is defined differently among surveys, depending on several factors, including, for example, the conceptual model of disability the survey designer uses (12). The NHIS includes many questions relating to structural and functional impairments and activity limitations, enabling investigators to achieve greater detail in defining disability. The Nagi model of disability considers the causes of disability to be multidimensional and to include individual attributes and environment (13). The Nagi model was a cornerstone of the 1991 Institute of Medicine report on disability (5), which was published the year following passage of the Americans with Disabilities Act (ADA) (14). The ADA defines disability as a physical or mental impairment that substantially limits 1 or more major life activities (14). Consistent with the ADA definition of disability, the NHIS questions were used to construct 2 disability subcategories: basic actions difficulty and complex activity limitation (10).

Basic actions are essential functions that enable a person to maintain independence and participate in social activities (10). Basic actions difficulties include movement, emotional, sensory, and cognitive difficulties. Complex activity limitations are complications experienced in performing tasks or engaging in social actions (5,10). The components of complex activity limitation include social and work limitations, as well as limitations with self-care activities of daily living (ADL) or instrumental activities of daily living (IADL). NHIS used several questions to define the components of basic actions difficulty and complex activity limitation. Respondents who were identified as having a basic actions difficulty or complex activity limitation were classified as having any limitation. These disability subcategories and their various components were used both separately and collectively to assess the association between BMI category and disability.

### Statistical analyses

We used SAS-callable SUDAAN, version 10.0.1 (Research Triangle Institute, Research Triangle Park, North Carolina) to obtain national estimates of disability, type of disability, and obesity prevalence. We obtained prevalence estimates of sociodemographic variables to examine the population distribution of obese adults compared with those who are not obese. We examined the following variables: sex, age group (18–44 y, 45–64 y, and ≥65 y), race/ethnicity (Hispanic, non-Hispanic white, non-Hispanic black, non-Hispanic of another race ), education level (less than a high school education, high school graduate, associate or technical degree, college graduate), employment status (employed, unemployed, retired/student/homemaker, unable to work), annual household income (<$35,000, $35,000–$74,999, ≥$75,000), marital status (married, living with partner, widowed, divorced/separated, never married), and region of the country. We combined data from multiple years to ensure that stable estimates were calculated for the various types of disability. Because adjusted measures of obesity have not shown significant change from 2003 to 2010 (1), the survey years of 2003 through 2009 were combined. A total of 190,786 respondents completed the sample adult questionnaire from 2003 through 2009, yielding an aggregate final sample adult response rate of 69.0% (the individual year final sample adult response rates ranged from 62.6% to 74.2%). A total of 178,999 respondents were included in our overall analysis. For each of the BMI categories, our total sample sizes were underweight (n = 3,182); normal weight (n = 66,698); overweight (n = 63,510); and obese (n = 45,609). Respondents were excluded from the entire analysis if their BMI information was missing (n = 8,645), if they had an extreme BMI value (<15 or >50 [ n = 1,173]), or if they were pregnant (n = 2,092). Exclusions were not mutually exclusive, so there was some overlap among categories. Data were weighted to account for differential probability of selection and to adjust for nonresponse. Estimates were age-adjusted to the 2000 US standard population (15) to account for the higher prevalence of disability in older age groups (10). We conducted *t* tests to compare the prevalence of disability in the weight status categories of underweight, overweight, and obese to prevalence in the normal-weight category for each disability type, by sex.

## Results

In our analytic sample, 1.9% of adults were underweight, 37.8% were normal weight, 35.3% were overweight, and 25.0% were obese. BMI varied by sex and by race/ethnicity (Tables 1 and 2). We found that 1.0% of men and 2.8% of women were underweight, 31.1% of men and 44.6% of women were normal weight, 42.7% of men and 28.0% of women were overweight, and 25.3% of men and 24.6% of women were obese.

**Table 1:** Demographic Characteristics of Adult Men,^a^ by Weight Status,^b^ 2003–2009 National Health Interview Survey^c^

Demographic Characteristic	Total, % (95% CI)	Underweight, % (95% CI)	Normal Weight, % (95% CI)	Overweight, % (95% CI)	Obese, % (95% CI)
**Age, y**					
18–44	51.6 (51.2–52.1)	65.0 (60.4–69.4)	58.7 (58.0–59.5)	48.6 (47.9–49.3)	47.8 (46.9–48.6)
45–64	33.9 (33.4–34.3)	16.0 (13.0–19.4)	26.2 (25.5–26.8)	36.2 (35.6–36.8)	39.9 (39.1–40.7)
≥65	14.5 (14.2–14.8)	19.0 (15.9–22.5)	15.1 (14.6–15.6)	15.2 (14.8–15.7)	12.4 (11.9–12.9)
**Race/ethnicity**					
Hispanic	13.7 (13.4–14.1)	8.5 (6.5–11.1)	11.6 (11.2–12.1)	14.9 (14.4–15.4)	14.8 (14.2–15.4)
Non–Hispanic white	70.6 (70.1–71.0)	67.6 (63.0–71.8)	70.1 (69.4–70.8)	70.9 (70.3–71.6)	70.0 (69.2–70.8)
Non–Hispanic black	10.6 (10.3–10.9)	11.6 (9.0–14.8)	10.4 (10.0–10.9)	9.8 (9.4–10.2)	12.4 (11.8–13.0)
Other race, non–Hispanic	5.1 (4.9–5.3)	12.3 (9.5–15.7)	7.8 (7.4–8.2)	4.4 (4.1–4.7)	2.8 (2.5–3.1)
**Education level**					
Less than high school	16.7 (16.4–17.1)	29.7 (24.9–35.0)	17.5 (16.9–18.1)	15.4 (14.9–15.9)	17.5 (16.9–18.2)
High school graduate	47.7 (47.3–48.2)	50.9 (45.8–56.1)	46.1 (45.3–46.9)	46.0 (45.4–46.7)	52.1 (51.2–53.0)
Associate/technical degree	8.8 (8.5–9.0)	4.1 (2.8–6.0)	7.6 (7.2–8.1)	9.4 (9.0–9.8)	9.4 (8.9–9.9)
College graduate	26.8 (26.3–27.2)	15.3 (11.8–19.6)	28.8 (28.1–29.5)	29.2 (28.5–29.8)	21.0 (20.2–21.7)
**Employment status**					
Employed	69.7 (69.3–70.0)	48.7 (43.8–53.6)	66.8 (66.1–67.4)	72.5 (72.0–73.0)	69.5 (68.8–70.1)
Unemployed	5.2 (5.0–5.4)	9.3 (6.8–12.6)	5.7 (5.3–6.0)	4.7 (4.4–5.0)	5.2 (4.9–5.6)
Retired/student/homemaker	19.3 (19.0–19.6)	26.5 (23.0–30.4)	21.6 (21.1–22.2)	18.2 (17.8–18.6)	17.4 (17.0–17.9)
Unable to work	5.9 (5.7–6.1)	15.5 (12.3–19.3)	5.9 (5.6–6.3)	4.7 (4.4–4.9)	7.9 (7.5–8.3)
**Income, $**					
<35,000	32.8 (32.3–33.2)	53.4 (48.0–58.7)	37.4 (36.6–38.3)	30.0 (29.4–30.7)	31.0 (30.2–31.8)
35,000–74,999	35.5 (35.0–36.0)	25.5 (21.0–30.5)	33.1 (32.3–33.9)	35.8 (35.1–36.5)	38.4 (37.5–39.3)
≥75,000	31.8 (31.2–32.3)	21.1 (16.4–29.9)	29.4 (28.6–30.3)	34.2 (33.5–34.9)	30.6 (29.7–31.5)
**Marital status**					
Married	58.6 (58.2–59.1)	35.0 (30.9–39.2)	50.3 (49.5–51.0)	62.4 (61.8–63.1)	63.9 (63.1–64.7)
Widowed	2.8 (2.7–2.9)	3.9 (2.8–5.5)	3.4 (3.2–3.6)	2.6 (2.4–2.7)	2.4 (2.2–2.6)
Divorced/separated	9.0 (8.8–9.2)	8.8 (6.7–11.6)	9.4 (9.0–9.7)	9.0 (8.7–9.3)	9.0 (8.6–9.4)
Never married	23.0 (22.6–23.4)	46.9 (43.2–50.7)	30.2 (29.5–30.8)	19.5 (18.9–20.0)	18.1 (17.5–18.7)
Living with partner	6.6 (6.4–6.8)	5.3 (3.5–7.9)	6.8 (6.5–7.2)	6.5 (6.2–6.9)	6.6 (6.2–7.0)
**Region**					
Northeast	17.1 (16.6–17.6)	16.3 (12.2–21.5)	17.5 (16.7–18.2)	17.4 (16.7–18.0)	16.3 (15.6–17.0)
Midwest	24.4 (23.8–25.0)	22.1 (17.8–27.2)	23.4 (22.6–24.2)	24.0 (23.3–24.8)	26.1 (25.1–27.0)
South	36.4 (35.7–37.0)	40.5 (35.0–46.3)	36.0 (35.1–36.9)	35.7 (34.9–36.6)	37.8 (36.7–38.8)
West	22.2 (21.6–22.7)	21.0 (17.3–25.3)	23.2 (22.4–24.0)	22.9 (22.2–23.5)	19.9 (19.1–20.7)

Abbreviation: CI, confidence interval; BMI, body mass index.

a Age adjusted to the 2000 US standard population (15).

b Excludes those respondents with extreme BMI (calculated as weight in kilograms divided by the square of height in meters [kg/m^2^] values (<15 and >50). BMI categories defined as follows: underweight, BMI <18.5; normal weight, BMI of 18.5-24.9; overweight, BMI of 25.0-29.9; obese, BMI ≥30.

c Because of rounding, columns may not add to 100%.

**Table 2:** Demographic Characteristics of Adult Women^a^ by Weight Status,^b^ 2003–2009 National Health Interview Survey^c^

Demographic Characteristic	Total, % (95% CI)	Underweight, % (95% CI)	Normal Weight, % (95% CI)	Overweight, % (95% CI)	Obese, % (95% CI)
**Age, y**					
18–44	48.0 (47.6–48.4)	60.1 (57.7–62.5)	53.9 (53.3–54.5)	42.4 (41.6–43.1)	42.7 (41.9–43.5)
45–64	33.6 (33.3–34.0)	19.7 (17.7–21.8)	29.1 (28.5–29.6)	36.3 (35.6–37.1)	40.1 (39.4–40.9)
≥65	18.4 (18.1–18.7)	20.2 (18.5–22.0)	17.0 (16.6–17.5)	21.3 (20.8–21.9)	17.2 (16.6–17.7)
**Race/ethnicity**					
Hispanic	12.4 (12.1–12.7)	6.4 (5.4–7.5)	10.0 (9.7–10.4)	15.2 (14.6–15.7)	14.4 (13.9–15.0)
Non–Hispanic white	69.9 (69.5–70.4)	74.6 (72.3–76.7)	74.8 (74.3–75.4)	67.0 (66.2–67.7)	63.4 (62.6–64.3)
Non–Hispanic black	12.5 (12.2–12.8)	6.9 (5.8–8.1)	8.2 (7.9–8.5)	13.8 (13.3–14.3)	19.7 (19.0–20.4)
Other race, non–Hispanic	5.2 (5.0–5.4)	12.2 (10.5–14.1)	6.9 (6.6–7.3)	4.1 (3.8–4.4)	2.4 (2.1–2.7)
**Education level**					
Less than high school	15.3 (15.0–15.6)	15.1 (13.2–17.2)	12.4 (12.0–12.8)	16.6 (16.0–17.1)	19.2 (18.6–19.8)
High school graduate	49.0 (48.6–49.4)	49.3 (46.7–51.9)	46.1 (45.5–46.7)	50.1 (49.3–50.9)	53.0 (52.2–53.8)
Associate/technical degree	10.6 (10.3–10.8)	8.6 (7.3–10.0)	10.0 (9.7–10.4)	11.0 (10.5–11.5)	11.4 (10.9–11.9)
College graduate	25.2 (24.8–25.6)	27.0 (24.8–29.3)	31.5 (30.9–32.1)	22.4 (21.7–23.0)	16.4 (15.9–17.0)
**Employment status**					
Employed	57.6 (57.2–58.0)	54.5 (52.1–56.8)	59.0 (58.4–59.6)	58.5 (57.8–59.2)	55.0 (54.2–55.7)
Unemployed	4.3 (4.2–4.5)	4.9 (3.9–6.1)	4.0 (3.8–4.3)	4.3 (3.9–4.6)	5.0 (4.7–5.4)
Retired/student/homemaker	31.6 (31.3–32.0)	32.3 (30.3–34.3)	32.7 (32.2–33.3)	31.6 (31.0–32.2)	29.2 (28.5–29.8)
Unable to work	6.4 (6.2–6.6)	8.4 (7.1–9.9)	4.2 (4.0–4.4)	5.7 (5.3–6.0)	10.9 (10.4–11.4)
**Income, $**					
<35,000	38.5 (38.0–39.0)	42.2 (39.5–44.9)	34.2 (33.6–34.9)	38.6 (37.8–39.4)	45.2 (44.4–46.1)
35,000–74,999	32.8 (32.3–33.2)	30.5 (27.5–33.6)	31.3 (30.7–31.9)	33.9 (33.1–34.6)	34.4 (33.6–35.2)
75,000	28.8 (28.3–29.2)	27.3 (24.7–30.1)	34.5 (33.8–35.2)	27.5 (26.7–28.3)	20.4 (19.6–21.2)
**Marital status**					
Married	53.4 (52.9–53.8)	44.9 (42.5–47.4)	54.0 (53.3–54.6)	55.8 (55.0–56.5)	51.3 (50.5–52.0)
Widowed	9.1 (8.9–9.3)	10.9 (10.0–11.9)	8.9 (8.7–9.2)	9.0 (8.8–9.3)	9.2 (8.9–9.5)
Divorced/separated	12.3 (12.0–12.5)	11.4 (10.0–13.1)	11.1 (10.8–11.4)	12.5 (12.1–12.9)	14.5 (14.0–15.0)
Never married	19.2 (18.8–19.5)	26.1 (24.0–28.3)	19.8 (19.3–20.3)	16.7 (16.1–17.3)	19.2 (18.5–19.8)
Living with partner	6.1 (5.9–6.3)	6.6 (5.5–7.9)	6.2 (5.9–6.6)	6.0 (5.6–6.4)	5.9 (5.5–6.3)
**Region**					
Northeast	18.0 (17.6–18.5)	18.5 (16.2–21.1)	19.2 (18.6–19.8)	17.8 (17.1–18.5)	15.9 (15.3–16.6)
Midwest	24.1 (23.5–24.7)	24.0 (21.7–26.4)	23.6 (22.8–24.3)	23.8 (23.1–24.7)	25.2 (24.3–26.1)
South	36.8 (36.2–37.4)	35.7 (33.0–38.4)	34.7 (34.0–35.5)	37.4 (36.5–38.3)	40.1 (39.1–41.2)
West	21.1 (20.6–21.6)	21.8 (19.4–24.5)	22.5 (21.8–23.1)	20.9 (20.2–21.7)	18.8 (18.0–19.6)

Abbreviation: CI, confidence interval; BMI, body mass index.

a Age adjusted to the 2000 US standard population (15).

b Excludes those respondents with extreme BMI (calculated as weight in kilograms divided by the square of height in meters [kg/m^2^] values (<15 and >50). BMI categories defined as follows: underweight, BMI <18.5; normal weight, BMI of 18.5–24.9; overweight, BMI of 25.0–29.9; obese, BMI ≥30.

c Because of rounding, columns may not add to 100%.

### Prevalence of disability by BMI category

Overall, 31.2% of adults self-reported a disability, approximately 27.0% of normal-weight adults and 41.0% of obese adults (any limitation). Among men, 26.7% of those at a normal weight had a disability compared with 35.2% of those who were obese (Table 3). Among women, 26.8% of those at a normal weight had a disability compared with 46.9% of those who were obese (Table 4). Underweight men and women were more likely to have a disability (any limitation) than those of normal weight (Tables 3 and 4).

**Table 3:** Disability Prevalence Estimates of Men^a^ (N = 81,363), Overall and by Weight Status,^b^ 2003–2009 National Health Interview Survey

Disability^c^	Total, % (95% CI)	Underweight, % (95% CI)	Normal Weight, % (95% CI)	Overweight, % (95% CI)	Obese, % (95% CI)
**Basic actions difficulty**	26.4 (26.0–26.8)	39.7 (35.3–44.2)	24.1 (23.5–24.7)	23.9 (23.4–24.4)	32.9 (32.2–33.7)
Movement	18.4 (18.0–18.7)	28.1 (24.2–32.3)	15.5 (15.0–16.0)	16.1 (15.6–16.5)	25.3 (24.7–26.0)
Sensory	12.5 (12.2–12.8)	18.0 (14.7–21.9)	12.2 (11.7–12.8)	11.4 (11.0–11.9)	14.3 (13.7–14.8)
Visual	8.0 (7.8–8.2)	13.5 (10.5–17.1)	8.1 (7.7–8.6)	7.2 (6.8–7.5)	9.1 (8.6–9.6)
Hearing	5.6 (5.4–5.8)	7.2 (5.1–10.1)	5.2 (4.9–5.6)	5.3 (5.0–5.5)	6.5 (6.1–7.0)
Emotional	2.3 (2.2–2.5)	7.0 (4.9–9.9)	2.1 (1.9–2.3)	1.9 (1.7–2.1)	3.1 (2.8–3.4)
Cognitive	2.8 (2.7–3.0)	9.2 (7.0–12.0)	3.3 (3.1–3.6)	2.3 (2.1–2.5)	2.9 (2.7–3.2)
**Complex activity limitation**	13.2 (12.9–13.5)	28.2 (24.4–32.3)	13.4 (12.9–13.9)	10.8 (10.4–11.2)	16.7 (16.1–17.3)
Self–care	3.2 (3.0–3.4)	12.1 (9.4–15.5)	3.7 (3.5–4.0)	2.4 (2.2–2.6)	3.6 (3.3–3.9)
ADL	1.5 (1.4–1.6)	6.8 (4.7–9.8)	1.8 (1.6–2.0)	1.0 (0.9–1.1)	1.7 (1.5–1.9)
IADL	2.9 (2.7–3.0)	10.6 (8.2–13.5)	3.4 (3.1–3.7)	2.1 (2.0–2.3)	3.1 (2.9–3.4)
Social	5.8 (5.6–6.0)	14.6 (11.5–18.3)	5.8 (5.4–6.1)	4.5 (4.3–4.8)	7.7 (7.2–8.1)
Work	11.1 (10.8–11.3)	24.8 (21.1–28.8)	11.3 (10.8–11.7)	9.0 (8.6–9.3)	14.1 (13.5–14.6)
Any limitation	28.7 (28.3–29.0)	42.6 (38.3–47.0)	26.7 (26.1–27.4)	25.9 (25.3– 26.4)	35.2 (34.4–35.9)
No limitation	71.3 (71.0–71.7)	57.4 (53.0–61.7)	73.3 (72.6–73.9)	74.1 (73.6–74.7)	64.8 (64.1–65.6)

Abbreviation: CI, confidence interval; ADL, activities of daily living; IADL, instrumental activities of daily living.

a Age adjusted to the 2000 US standard population (15).

b Excludes those respondents with extreme BMI (calculated as weight in kilograms divided by the square of height in meters [kg/m^2^] values (<15 and >50). BMI categories defined as follows: underweight, BMI <18.5; normal weight, BMI of 18.5–24.9; overweight, BMI of 25.0–29.9; obese, BMI ≥30.

c Disability groups are not mutually exclusive and respondents may be represented in more than 1 type of disability.

**Table 4:** Disability Prevalence Estimates of Women,^a^ (N = 97,636), Overall and by Weight Status,^b^ 2003–2009 National Health Interview Survey

Disability^c^	Total, % (95% CI)	Underweight, % (95% CI)	Normal Weight, % (95% CI)	Overweight, % (95% CI)	Obese, % (95% CI)
**Basic actions difficulty**	31.9 (31.5–32.3)	31.2 (29.0–33.4)	25.1 (24.6–25.6)	30.6 (30.0–31.3)	45.0 (44.2–45.8)
Movement	24.6 (24.3–24.9)	22.4 (20.5–24.4)	17.6 (17.2–18.0)	23.4 (22.8–23.9)	37.9 (37.2–38.6)
Sensory	13.1 (12.8–13.3)	14.6 (13.0–16.4)	11.5 (11.1–11.9)	12.4 (11.9–12.9)	16.4 (15.8–16.9)
Visual	10.7 (10.5–11.0)	11.3 (9.9–12.9)	9.3 (9.0–9.7)	10.1 (9.7–10.6)	13.8 (13.3–14.4)
Hearing	3.2 (3.1–3.3)	4.6 (3.7–5.7)	2.9 (2.7–3.1)	3.1 (2.8–3.3)	3.6 (3.4–3.9)
Emotional	3.7 (3.5–3.8)	5.2 (4.2–6.4)	2.6 (2.4–2.8)	3.2 (3.0–3.5)	5.9 (5.6–6.3)
Cognitive	3.0 (2.9–3.1)	5.1 (4.3–6.1)	2.6 (2.4–2.8)	2.6 (2.4–2.8)	4.0 (3.7–4.3)
**Complex activity limitation**	15.3 (15.1–15.6)	19.4 (17.6–21.3)	11.6 (11.3–12.0)	13.8 (13.4–14.3)	23.0 (22.4–23.7)
Self–care	4.9 (4.8–5.1)	9.5 (8.2–10.9)	4.0 (3.8–4.2)	4.0 (3.8–4.3)	7.2 (6.8–7.6)
ADL	2.1 (2.0–2.2)	5.0 (4.1–6.2)	1.7 (1.6–1.9)	1.7 (1.5–1.8)	2.9 (2.7–3.2)
IADL	4.6 (4.5–4.8)	8.8 (7.7–10.2)	3.8 (3.6–4.0)	3.7 (3.5–4.0)	6.7 (6.4–7.1)
Social	8.5 (8.3–8.8)	11.4 (9.9–13.0)	6.2 (5.9–6.5)	7.4 (7.1–7.8)	13.5 (13.0–14.0)
Work	11.9 (11.6–12.1)	14.8 (13.2–16.5)	8.9 (8.6–9.2)	10.6 (10.2–11.0)	18.2 (17.6–18.7)
Any limitation	33.6 (33.3–34.0)	33.8 (31.6–36.1)	26.8 (26.3–27.3)	32.3 (31.7–32.9)	46.9 (46.1–47.6)
No limitation	66.4 (66.0–66.7)	66.2 (63.9–68.4)	73.2 (72.7–73.7)	67.7 (67.1–68.3)	53.1 (52.4–53.9)

Abbreviation: CI, confidence interval; ADL, activities of daily living; IADL, instrumental activities of daily living.

a Age adjusted to the 2000 US standard population (15).

b Excludes those respondents with extreme BMI (calculated as weight in kilograms divided by the square of height in meters [kg/m^2^] values (<15 and >50) and pregnant women. BMI categories defined as follows: underweight, BMI <18.5; normal weight, BMI of 18.5–24.9; overweight, BMI of 25.0–29.9; obese, BMI ≥30.

c Disability groups are not mutually exclusive and respondents may be represented in more than 1 type of disability.

### Basic actions difficulty

Among men, 24.1% of those at a normal weight reported having a basic-actions difficulty compared with 32.9% of those who were obese. Among women, 25.1% of those at a normal weight had a basic-actions difficulty compared with 45.0% of those who were obese. Movement difficulty was the most common type of basic-actions difficulty, affecting 15.5% of normal-weight men, 25.3% of obese men, 17.6% of normal-weight women, and 37.9% of obese women. Emotional difficulty was the least common type of basic-actions difficulty that normal-weight men and women reported (2.1% and 2.6%, respectively). In contrast, cognitive difficulties were the least common disability among obese men, affecting 2.9%, and hearing difficulties were the least common disability among obese women, affecting 3.6%.

The prevalence of movement difficulty was approximately 1.5 times higher for obese men in comparison to normal-weight men (15.5% vs 25.3%; *P* < .001). Similarly, the prevalence of movement difficulty was twice as high for obese women as for normal-weight women (17.6% vs 37.9%; *P* < .001).

Underweight men were more likely than normal-weight men to experience basic-actions difficulty (39.7% vs 24.1%, *P* ,<.001) as were underweight women (31.2% vs 25.1%, *P* ≤ .001). Underweight men had a significantly higher prevalence of disability across all types of basic action difficulty than normal-weight men, with the exception of hearing difficulty, which was similar for both groups. Underweight women had a significantly higher prevalence of all basic-actions difficulties than normal-weight women.

### Complex activity limitation

Among men, 13.4% of those at a normal weight reported having a complex-activity limitation compared with 16.7% of those who were obese. Among women, 11.6% of those at a normal weight reported having a complex-activity limitation compared with 23.0% of those who were obese. Work limitation was the most common type of complex-activity limitation, affecting 11.3% and 8.9% of normal-weight men and women, respectively. It was the most common type of complex-activity limitation affecting obese adults. ADL limitation was the least common type of complex-activity limitation, affecting 1.8% and 1.7% of normal-weight men and women and 1.7% and 2.9% of obese men and women, respectively.

Underweight men were more likely than normal-weight men to experience complex-activity limitations (28.2% vs 13.4%, *P* ≤ .001) as were underweight women (19.4% vs 11.6%, *P* ≤ .001). Underweight men and men had significantly higher prevalence of complex-activity limitations than their normal-weight counterparts. (Tables 3 and 4).

## Discussion

To our knowledge, this work is among the first in the United States to include type of disability as a variable in describing the demographic characteristics of US adults by BMI. Our findings show that more than 40% of obese adults in our sample had at least 1 disability.

Excluding underweight respondents, disability prevalence increased among respondents as their BMI increased. Although this was noted for most types of disability, it was highest among those reporting a movement difficulty. Our finding that movement difficulty was substantially higher among those who are obese compared to those of normal weight is consistent with prior research that found that people who were obese were more likely than people of a normal weight to have a functional impairment (16) and to have an increased risk of ADL limitation (17). Movement difficulty may hinder physical activity, preventing people with disabilities from meeting the physical activity guidelines for adults with disabilities; this is problematic because engaging in physical activity is an aspect of weight loss or weight maintenance (18). The prevalence of visual limitation also increased as BMI increased. Some studies have linked obesity with certain eye disorders, although empirical evidence is mixed (19). However, visual difficulty may limit the ability to navigate environment, and adults with visual impairments report more difficulty with physical activity (20).

Obese adults also reported higher prevalence of social and work limitation compared with those of a normal weight. This limitation in work is consistent with prior research (21) that demonstrated that younger and middle-aged obese workers had a reported prevalence of work limitation similar to that of middle-aged and older-aged workers, respectively, who were not overweight or obese. Our study shows that the prevalence of work limitation increased 2.8 percentage points for obese men compared with normal-weight men (11.3% vs 14.1%) and 9.3 percentage points for obese women compared with normal-weight women (8.9% vs 18.2%). The reason behind the sex difference is unclear and a possible direction for future work.

We found a higher prevalence of disability among underweight men than among obese men. However, underweight women had a lower prevalence of disability than obese women. Among obesity categories by disability type, the prevalence of disability followed a reverse *J*- or *U*-shaped distribution for men and a *J*-shaped distribution for women. A *J*- or *U*-shaped distribution has been noted in work comparing BMI with death (22) and illness (23). The finding that people who are underweight have a higher prevalence of disability may not be unusual because people who are underweight are more likely than people who are overweight to report moderate to heavy levels of cigarette smoking (23), a leading cause of illness and death (24). Furthermore, being underweight has been associated with early mortality among people with cognitive impairments (25), and illness may cause a person to become underweight or to develop a disability.

We note several additional limitations to our analysis. First, our findings likely underestimate disability among people who are obese. That is, BMI may underestimate obesity (26) for people with certain disabilities related to differences in body composition, such as spinal cord injury (26) and limb loss (27). Alternative measures, such as measuring arm circumference (28), may be more appropriate for defining obesity in people with certain disabilities. Second, BMI measures in the NHIS are based on self-reported height and weight, which may underestimate obesity prevalence because of a possible reporting bias (29). Third, the results may be sensitive to the definition of disability used. That is, the disability definition used here is detailed, inclusive, and consistent with the definition used by ADA (10,14); thus, we believe that it is appropriate for public health purposes. However, if others were to use a more limited measure of disability, the findings may differ (eg, a measure of disability linked solely to the ability to work) (2). Fourth, we did not use the NHIS imputed income files to assess prevalence of adults in each household income category. Fifth, the NHIS does not survey institutionalized adults or those on active military duty; therefore, we may have underestimated the true prevalence of disability. Thus, our results cannot be generalized to these populations. Finally, obesity has been identified as a leading secondary condition experienced by people with a disability (6); also obesity may lead to disability. Addressing the issue of causality (ie, which came first, the obesity or the disability) requires information on the duration of obesity and disability. However, historical data on disability duration are largely unavailable. To reduce issues pertaining to causality, we excluded approximately 5% of respondents who reported weight as the cause of their disability. By re-estimating the data, we found our results were robust (ie, similar to those shown in Tables 3 and 4).

This research contributes to the literature on obesity prevalence by including disability as a demographic characteristic and considering type of disability in assessing the burden of obesity in a nationally representative US sample. People with disabilities comprise approximately 26.7% of the normal-weight adult population and 41.0% of obese adults. Knowing that a large percentage of people with obesity have a disability, and knowing the type of disability, will assist public health workers in designing interventions to reduce obesity that include people with disabilities. The systematic collection, analysis, and interpretation of surveillance data are essential to the planning, implementation, and evaluation of effective public health programs. Routine inclusion of disability as a variable in public health surveillance will inform and strengthen the planning and implementation of public health programs.

## Figures and Tables

**Table:** 

**1. The activity supported the learning objectives.**				
**Strongly Disagree**				**Strongly Agree**
1	2	3	4	5
**2. The material was organized clearly for learning to occur.**				
**Strongly Disagree**				**Strongly Agree**
1	2	3	4	5
**3. The content learned from this activity will impact my practice.**				
**Strongly Disagree**				**Strongly Agree**
1	2	3	4	5
**4. The activity was presented objectively and free of commercial bias.**				
**Strongly Disagree**				**Strongly Agree**
1	2	3	4	5

## Post-Test Information

To obtain credit, you should first read the journal article. After reading the article, you should be able to answer the following, related, multiple-choice questions. To complete the questions (with a minimum 70% passing score) and earn continuing medical education (CME) credit, please go to http://www.medscape.org/journal/pcd. Credit cannot be obtained for tests completed on paper, although you may use the worksheet below to keep a record of your answers. You must be a registered user on Medscape.org. If you are not registered on Medscape.org, please click on the "Register" link on the right hand side of the website to register. Only one answer is correct for each question. Once you successfully answer all post-test questions you will be able to view and/or print your certificate. For questions regarding the content of this activity, contact the accredited provider, CME@medscape.net. For technical assistance, contact CME@webmd.net. American Medical Association's Physician's Recognition Award (AMA PRA) credits are accepted in the US as evidence of participation in CME activities. For further information on this award, please refer to http://www.ama-assn.org/ama/pub/category/2922.html. The AMA has determined that physicians not licensed in the US who participate in this CME activity are eligible for **AMA PRA Category 1 Credits™**. Through agreements that the AMA has made with agencies in some countries, AMA PRA credit may be acceptable as evidence of participation in CME activities. If you are not licensed in the US, please complete the questions online, print the AMA PRA CME credit certificate and present it to your national medical association for review.

## Post-Test Questions

### Article Title: Effect of the Planet Health Intervention on Eating Disorder Symptoms in Massachusetts Middle Schools, 2005—2008

**CME Questions**

1. You are seeing a 48-year-old man with a body mass index (BMI) of 36 kg/m^2^. He says that he feels like he is "slowing down" overall and blames his obesity. Based on the results of the current study, what should you consider regarding the relationship between BMI and disability?
  - Over 40% of obese adults had at least 1 disability
  - Obesity affected the risk of disability among men, but not women
  - Obesity affected the risk of disability among women, but not men
  - There was no association between obesity and the rate of disability
2. As you get more details regarding this patient’s complaint, what should you consider was the most common type of basic actions difficulty among obese adults in the current study?
  - Movement difficulty
  - Emotional difficulty
  - Vision difficulty
  - Sensory difficulty
3. What was the most common complex activity limitation among obese adults in the current study?
  - Work limitation
  - Activities of daily living limitation
  - Social limitation
  - Self-care limitation
4. The patient asks you if becoming underweight (achieving a BMI of less than 18 kg/m^2^) would further reduce his basic and complex activity limitations. What can you tell him regarding the rate of disability associated with underweight status compared with disability associated with normal weight in the current study?
  - Underweight adults had the lowest risk of disability
  - Underweight status was associated with lower rates of basic actions difficulty only
  - Underweight adults had higher rates of basic actions difficulty and complex activity limitations than those of normal weight
  - Underweight status did not affect any risk of disability.

**Evaluation**
